# Nox1-Derived ROS Amplifies Calcium Entry and Enhances Pneumolysin-Induced Lung Endothelial Barrier Dysfunction in Hyperglycemia

**DOI:** 10.3390/antiox15030275

**Published:** 2026-02-24

**Authors:** Stephen Haigh, Feng Chen, Yanfang Yu, Zsuzsanna Bordan, Xueyi Li, Supriya Sridhar, Maritza J. Romero, Trinad Chakraborty, Gabor Csanyi, Austin T. Joshua, Tej V. Patel, Zachary L. Brown, Mitchel A. Shivers, Hunter G. Sellers, Farhana Ananna, Tohru Fukai, Masuko Ushio-Fukai, Eric J. Belin de Chantemele, Alexander Verin, David W. Stepp, Rudolf Lucas, David J. R. Fulton

**Affiliations:** 1 Vascular Biology Center, Augusta University, Augusta, GA 30912, USAzbordan@augusta.edu (Z.B.); ebelindechanteme@augusta.edu (E.J.B.d.C.); averin@augusta.edu (A.V.);; 2 Department of Forensic Medicine, Nanjing Medical University, Nanjing 211166, China; fchen@njmu.edu.cn (F.C.);; 3 Department of Genetics, Stanford University, Palo Alto, CA 94304, USA; 4 Institute for Medical Microbiology, Justus-Liebig University, Schubertstraße 81, 35392 Giessen, Germany; 5 Department of Pharmacology and Toxicology, Augusta University, Augusta, GA 30912, USA

**Keywords:** NOX1, obesity, lung, endothelial barrier, calcium, STIM1, TRPC1, mPTP

## Abstract

**Background:** *Streptococcus pneumonia* is the primary etiological agent of community-acquired pneumonia (CAP). Pneumococci promote severe lung injury through the release of virulence factors, including pneumolysin (PLY). Obesity/diabetes increases pneumonia-associated mortality, but the mechanisms remain elusive. We found that obese db/db mice have increased pulmonary barrier disruption to PLY. Previously we showed that upregulation of NOX1 in endothelial cells (EC) of db/db mice drives endothelial dysfunction, but a role for NOX1 in PLY-induced lung injury, especially in diabetic conditions, has not yet been described. **Results:** Increased NOX1 in lung ECs dose-dependently increased superoxide and EC barrier disruption (*p* < 0.05). Even at low activity levels, NOX1 greatly potentiated PLY-induced EC barrier disruption, whereas loss of NOX1 activity, either pharmacological or genetic, reduced barrier disruption (*p* < 0.05). Blockade of calcium entry protected the EC barrier from combined PLY and NOX1, indicating a key role for calcium. Hyperglycemia amplified PLY-enduced EC barrier disruption and intracellular calcium and these effects were mitigated by NOX1 inhibition and silencing (*p* < 0.05). NOX1-enhanced calcium entry was reduced by knockout of calcium sensor STIM1, and PLY-induced barrier disruption was reduced by STIM1 inhibition. Levels of STIM1, Orai1, TRPV4, or TRPC4 were unchanged by HG, but TRPC1 significantly increased (*p* < 0.05). NOX1 and HG promoted increased STIM1 and TRPC1 binding, and silencing TRPC1 ameliorated PLY-induced barrier disruption (*p* < 0.05). Increased calcium promoted mitochondrial permeability transition pore (MPTP) opening and PPIF inhibition protected EC barrier function (*p* < 0.05). **Conclusions:** These results suggest that elevated glucose levels in obesity primes EC barrier disruption by amplifying PLY-induced calcium influx via a novel NOX1, STIM1, TRPC1 and MPTP signaling axis.

## 1. Introduction

Pneumonia is a major health concern, contributing to ~2% of all deaths and is the major cause of death by infectious disease in children worldwide [1,2]. Pneumonia can result from a number of infectious agents, but one of the most prominent is *Streptococcus pneumoniae* which produces the virulence factor pneumolysin (PLY) [3]. PLY binds to cholesterol in the plasma membrane of targeted cells, resulting in self-assembly and pore formation. PLY facilitates the colonization, tissue invasion, and transmission of *Streptococcus pneumoniae* and is responsible for the resulting lung injury [4]. The severity of lung injury from pneumococcal infection is mainly caused by an impairment of the pulmonary vascular barrier function [5]. PLY disrupts the alveolar–capillary barriers and as such increases permeability and interstitial and alveolar fluid accumulation, resulting in impaired oxygen transport [6]. The ability of PLY to assemble pores in the cell membrane leads to changes in cell signaling and viability, but in particular it causes a large influx of extracellular calcium (Ca^2+^) [7,8]. Broad inhibition of calcium entry with lanthanum chloride, which blocks calcium channels via steric hindrance, surprisingly reduced PLY-induced barrier disruption in human lung microvascular endothelial cell (HLMVEC) monolayers [8]. Lanthanum chloride also protects against calcium entry in response to a related pore-forming toxin from *Listeria monocytogenes*, listeriolysin O [9]. The requirement for calcium channels as drivers of the loss in endothelial barrier function, despite pore formation, is surprising. The goal of this study was therefore to investigate the molecular mechanisms by which calcium entry compromises the pulmonary endothelial barrier.

STIM1 (stromal interaction molecule 1) is a Ca^2+^-sensitive protein found within the endoplasmic reticulum (ER), where it “senses” Ca^2+^ levels via its Ca^2+^-binding EF hand domains [10,11]. When Ca^2+^ is depleted in the ER via Ca^2+^ efflux, STIM1 activates ORAI1 ion channels leading to an influx of Ca^2+^ into the cell. This process is known as store-operated calcium entry. TRPC1 is another important channel in this process that is activated by STIM1 and has been shown to regulate barrier function in endothelial cells [12]. The influx of Ca^2+^ has many effects on endothelial cells [13]. Some of these effects are beneficial, such as the release of nitric oxide, but highly elevated calcium can impact endothelial cell function and viability, in part via the mitochondrial permeability transition pore (mPTP) [14]. The mPTP is a Ca^2+^-dependent pore in the inner mitochondrial membrane that increases the permeability of the inner membrane to solutes and small molecules [15]. The physiological basis for pore opening remains incompletely understood, but this process has been shown, pathologically, to be involved in cell dysfunction and death [16], effects similar to those elicited by PLY. The mPTP is a complex of proteins that was initially challenging to identify [17]. One of the major components is a protein called peptidyl-prolyl cis-trans isomerase F (PPIF), a protein chaperone involved in protein folding and assembly that is also integral to the formation of the mPTP [18]. Reactive oxygen species (ROS) have been shown to activate the mPTP, particularly following ischemia–reperfusion [19], but opening of the pore can also increase ROS [20]. PLY also elevates ROS, which contribute to the loss of barrier function.How elevated ROS impacts the ability of PLY to increase calcium and disrupt the endothelial barrier remains poorly understood and is a goal of this study.

Diabetes mellitus type 2 (T2D) is a major and growing health concern worldwide that confers an increased risk and severity of many diseases, including pneumonia [21]. Recent clinical studies have shown that diabetes significantly increases the risk of hospitalization and death from pneumonia; furthermore, the severity of disease correlates best with impaired metabolic control [22,23,24,25,26]. With rates of T2D increasing worldwide and with pneumococci representing a major etiological agent of bacterial pneumonia, understanding the mechanisms through which hyperglycemia affects the disruption of the EC barrier induced by the main pneumococcal virulence factor PLY is critical to foster the development of novel therapies, especially for diabetic patients with pneumonia. T2D risk factors include genetic, metabolic, and environmental components; however, the modifiable risk factors of obesity, such as low physical activity and an unhealthy diet also correlate with the rise in cases. The db/db obese mouse strain is a long-standing model of obesity and diabetes with significant microvascular disease [27,28]. A major cause of microvascular endothelial dysfunction in obese mice and humans is increased superoxide production and, in particular, increased expression/activity of NOX1 [28,29,30,31,32,33,34,35,36,37,38,39]. Superoxide is a reactive species (ROS) with deleterious actions in mouse models of obesity and diabetes with well-reported vascular deficits, mediated at least in part through its rapid, diffusion-limited reaction with nitric oxide. Superoxide levels are increased in biological systems through the actions of oxidant-generating enzymes, including the NADPH oxidase family [40], and through mitochondrial activity [41], and levels can be further impacted by the status of antioxidant molecules and enzyme systems. Several studies, including our own, have shown that depletion or inhibition of the superoxide-generating enzyme NADPH oxidase 1 (NOX1) can ameliorate these deficits [29,30,31,32,33,34,35,36,37,38,39,42]. NOX1 is a member of the NADPH oxidase (NOX) family of enzymes, and its activity is regulated by the co-expression of subunits NOXO1 and NOXA1. NOX1 converts NADPH to NADP+ and oxygen to superoxide [40]. Increased levels of superoxide have long been hypothesized to affect pathological changes in hyperglycemic conditions. However, it remains unknown whether changes in NOX1 activity and ROS levels, such as that seen in conditions of elevated glucose, impact PLY-induced EC barrier dysfunction and this was a major focus of our study.

## 2. Materials and Methods

This study investigated the mechanisms by which diabetes modulates lung endothelial barrier function, which involved both in vitro experiments using human endothelial cells and in vivo experiments using mouse models.

### 2.1. Cell Culture and Transfection

COS-7 and HEK-293 cells (ATCC) were grown in Dulbecco’s modified Eagle’s medium (DMEM) containing 100 U/mL penicillin, 100 mg/mL streptomycin, and 10% FBS. These cell lines were selected because of their relative ease of transfection, utility in select functional assays, and ability to survive single-cell isolation. Results were confirmed in endothelial cells. Human lung microvascular endothelial cells (HLMVECs) and human pulmonary artery endothelial cells (HPAECs) were isolated and grown in-house as previously described [43,44] or purchased from Lonza and grown in Endothelial Growth Medium-2-Microvessel (EGM-2MV) or EGM-2 (HPAECs) containing the requisite growth factors and 5% FBS (Lonza Walkersville, Inc., Walkersville, MD, USA). The cells were grown in a 5% CO_2_ incubator at 37 °C and used from passage 2–8. For siRNA transfection, HLMVECs were transfected using Lipofectamine RNAIMAX (Invitrogen, Carlsbad, CA, USA). The STIM1 KO HEK293 cells were created using the LentiCRISPR v2 system (Feng Zhang, Addgene plasmid # 52961; http://n2t.net/addgene:52961 (1 January 2026); RRID: Addgene_52961, Watertown, MA, USA) by single-cell isolation of KO colonies. 

### 2.2. DNA Constructs, Adenovirus, Antibodies, and Reagents

The STIM1 guides were made using the LentiCRISPR v2 system (Addgene plasmid # 52961). Guides targeting exon 1 of STIM1 (GCTGCTCGCCGCTCTTCGG) were inserted into the all-in-one vector. Lentivirus was made using a PEG-it Virus Precipitation Solution (SBI). SiRNA: NOX1, STIM1, and TRPC1 Thermo-Fisher Scientific (Waltham, MA, USA). Antibodies: anti-NOX1 Millipore-Sigma (Burlington, MA, USA) (SAB4200097), anti-GAPDH Thermo-Fisher (AM4300), anti-STIM1 Cell Signaling Technology (Danvers, MA, USA) (4149S), anti-TRPC1 Santa Cruz Biotechnology (Dallas, TX, USA) (sc-133076), anti-PO_4_-p38 Cell-Signaling Technology (9211S). NOX1, NOX01, NOXA1, and NOX4 adenovirus were purified using a Takara Adeno-X™ Maxi Purification Kit (Takara Bio, San Jose, CA, USA) [45]. The lanthanum chloride (CAS: 955272-06-7), glucose (CAS: 50-99-7) and mannitol (CAS: 69-65-8) were from Fisher Scientific (Waltham, MA, USA). The GKT136901 (CAS: 955272-06-7), TRO19622 (CAS: 22033-87-0), and YM-58483 (CAS: 223499-30-7) specimens were from Cayman Chemicals (Ann Arbor, MI, USA). The catalase was from Sigma-Aldrich (Saint Louis, MO, USA).

### 2.3. Endothelial Permeability

Transendothelial resistance (TER) was measured in confluent monolayers of HLMVECs or HPAECs. Acute changes in TER reflect changes in cell permeability [46]. In brief, approximately 60,000 HLMVECs/well were seeded into 8W10E arrays. The media were replaced with fresh media at 24 h. The process was repeated with serum-free media at 48 h immediately prior to the addition of PLY. The ECIS Zh model was used to monitor the resistance values of cells over time and was then normalized to time = 0. PLY or a vehicle was added after the resistance was stable, around 1200 ohms at a frequency of 4000 Hz, and the capacitance was between 22 and 29 nanoFarads.

### 2.4. Measurements of Intracellular Ca^2+^

Intracellular calcium levels were monitored using the calcium-activated photoprotein aequorin as described [47]. In brief, HLMVECs were transduced with an adenovirus encoding aequorin at 30 MOI, and 24 h later the aequorin was reconstituted by treating cells with 5 μM coelenterazine for 30 min in serum-free and phenol-free DMEM. The cells were then exposed to PLY, and the luminescence was recorded using a PolarSTAR luminometer (BMG Labtech, Ortenberg, Germany).

### 2.5. Measurements of Lung Barrier Function in Mice

All experiments involving animals were conducted with Institutional Animal Care and Use Committee approval (protocol # 2017-0919, 24 August 2023) and conformed with the National Institutes of Health Guide for the Care and Use of Laboratory Animals. Control and leptin-deficient animals were purchased from Jackson Labs; strain numbers 000664 (control) 000697 (leptin-deficient) and used at 15–16 wks of age. Intratracheal instillation of PLY (30 ng/mouse) was performed as described in [48] and 23 h later mice were injected IV with Evan’s blue dye (EBD) via the jugular vein. At 24 h post PLY injection, the mice were anesthetized, and their lungs were collected. The concentration of EBD extravasated was calculated using a standard curve of ug of EBD per g of wet lung as described [49].

### 2.6. Statistical Analysis

Data is expressed as means ± SE, and statistical analyses were performed using GraphPad software 10 (GraphPad Software Inc., San Diego, CA, USA) with a two-tailed Student’s *t*-test or ANOVA with a post hoc test where appropriate. Statistical significance was considered as *p* < 0.05.

## 3. Results

### 3.1. Lung Capillary Leak Is Significantly Increased in Obese, T2D Mice in Response to the Bacterial Toxin PLY

**Figure 1:**
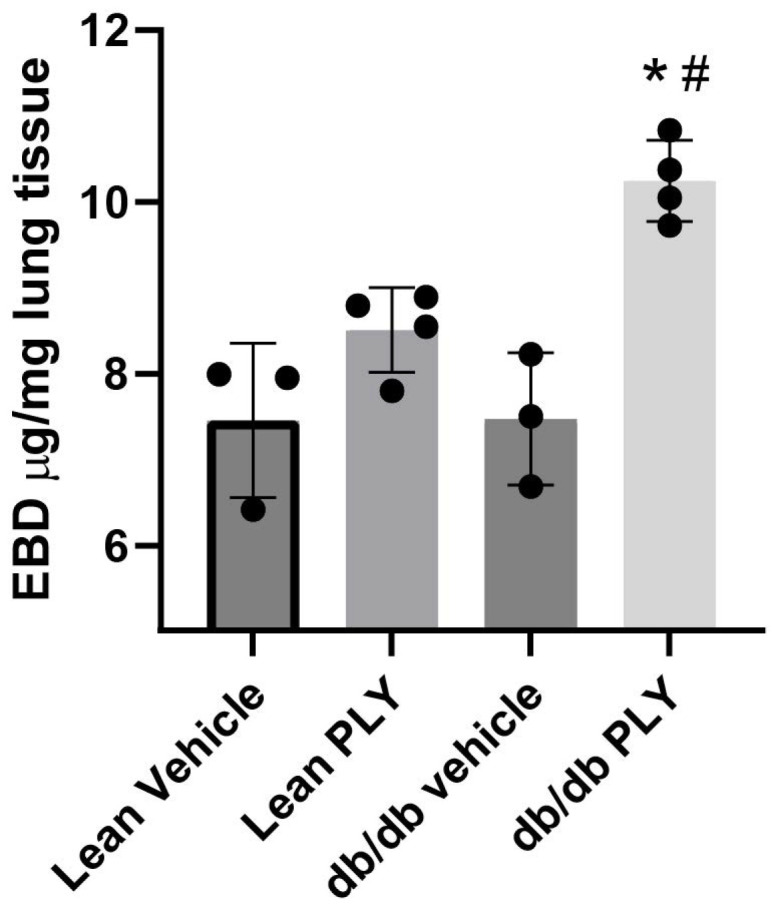
**Increased sensitivity to PLY-induced lung barrier disruption in the db/db mouse model of T2D.** Lean and obese (db/db) 16 wk old mice were exposed to 30 ng PLY delivered IT and vascular permeability was assessed by the Evan’s blue dye incorporation method as shown previously [8,48]. Data is expressed as means ± S.E.; * *p* < 0.05 vs. lean PLY; # *p*< 0.05 vs. db/db vehicle; ANOVA; *n* = 3–4.

Sixteen-week-old male leptin-receptor-deficient db/db mice (mean blood glucose level: 174.6 + 7.86 mg/dL) were used as a model of obesity and T2D and were compared to lean controls (mean blood glucose level: 121.4 + 11.9 mg/dL; *p* < 0.01 vs. db/db). As shown in Figure 1, we observed significantly increased pulmonary vascular leakage as determined by Evan’s blue dye incorporation into lung tissue in db/db mice in response to i.t. administration of a low dose of PLY, as compared to age-matched male lean mice (30 ng PLY/mouse; *n* = 4 per group; *: *p* < 0.05). Since the db/db mice had a significantly higher body weight (40.97 ± 4.88 g) than the lean mice (26.7 ± 1.40 g), this observation is all the more notable, as we used an equal dose of PLY (30 ng/mouse) that was not based on body weight.

### 3.2. High Glucose Increases PLY-Induced Endothelial Barrier Disruption Through NOX1 Upregulated Ca^2+^ Influx

**Figure 2:**
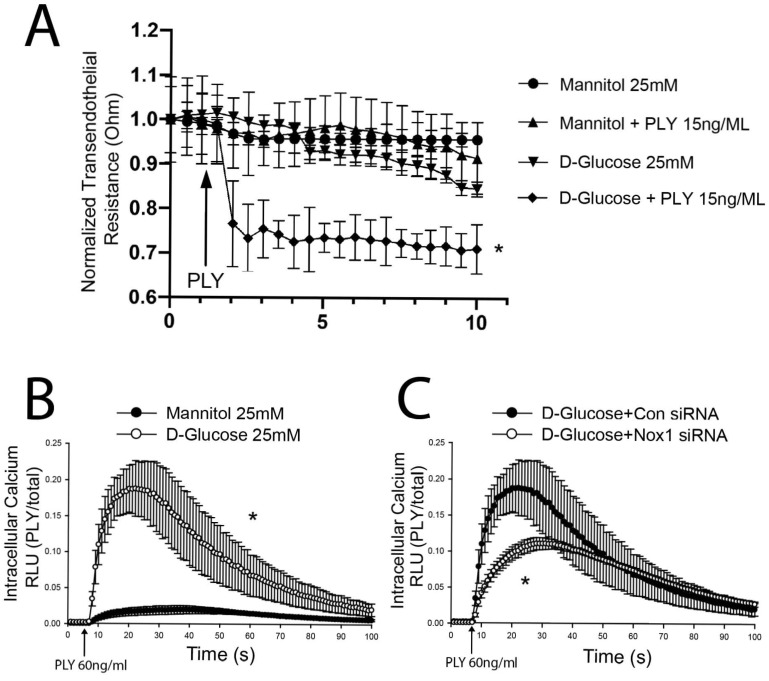
**HG treatment increases sensitivity to PLY-induced calcium entry in HLMVECs in an NOX1-dependent manner.** (**A**) HLMVECs were exposed to high concentrations of glucose (HG, 25 mM for 48 h) or the osmotic control mannitol (25 mM, 48 h) and challenged with a subthreshold concentration of PLY (15 ng/mL) and endothelial barrier disruption was assessed. *n* = 4. (**B**) HLMVECs were incubated for 48 h in HG (25 mM) or control (mannitol) conditions and then calcium influx was measured using adAEQ. *n* = 8. (**C**) HG-treated cells were transfected with control and NOX1 siRNA and then treated with PLY, and calcium influx was measured using ad-AEQ. *n* = 8. Data is expressed as means ± S.E.; * *p* < 0.05 vs. control; Student’s *t*-test of peak response.

A key component in the pathology of obesity-associated diabetes is endothelial dysfunction, wherein a major contributor is hyperglycemia. This can be mimicked in vitro by high-glucose (HG) treatment using 25 mM D-glucose. To test the effect of HG treatment on PLY-induced barrier disruption, HLMVECs were treated with either D-glucose or mannitol (iso-osmotic control) for 48 h before a subthreshold PLY (15 ng/mL) treatment. PLY-induced barrier disruption was significantly exacerbated by HG treatment (Figure 2A). A major aspect of PLY-induced cell injury is pore-induced Ca^2+^ influx [6]. To test whether HG treatment potentiated PLY-induced Ca^2+^ influx, intracellular Ca^2+^ levels were measured via adenoviral overexpression of the calcium-dependent luminescent protein aequorin. Aequorin reacts with coelenterazine to make light in the presence of Ca^2+^. High-glucose treatment of HLMVECs dramatically increased the amount of Ca^2+^ influx after PLY treatment (Figure 2B). To determine the effects of NOX1 expression on Ca^2+^ influx, NOX1-specific, but not scrambled, control siRNA-treated HLMVECs showed decreased Ca^2+^ influx in HG conditions (Figure 2C). In summary, these results are consistent with the ability of HG treatment to amplify PLY-induced EC barrier disruption in vivo and in vitro and to increase PLY-induced increases in intracellular calcium in an NOX1-dependent manner.

### 3.3. Increased NOX1 Activity Promotes PLY-Induced Barrier Dysfunction in Human Endothelial Cell Monolayers

**Figure 3:**
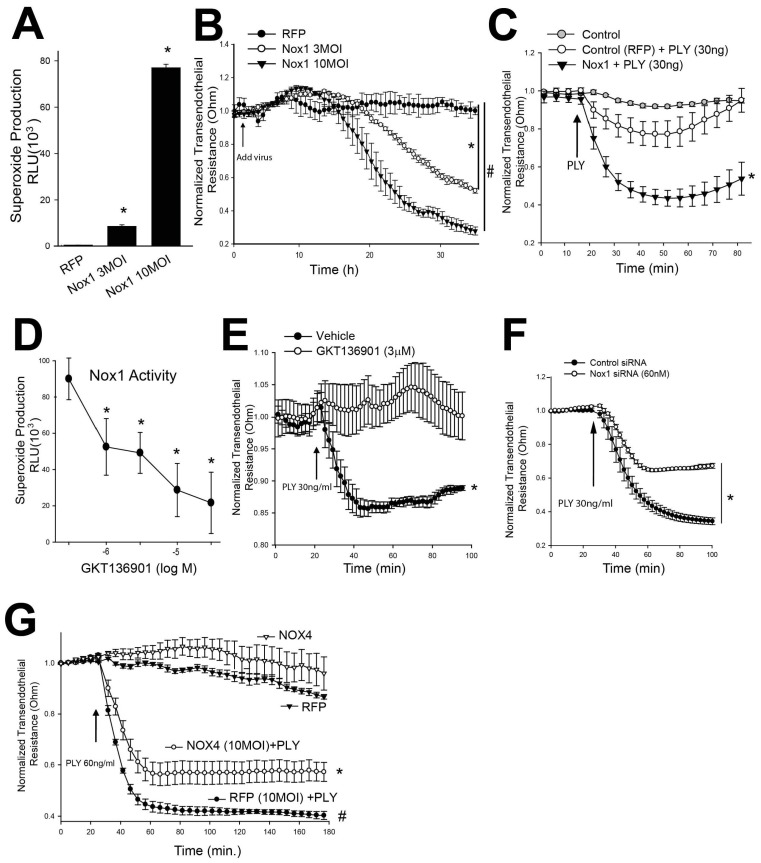
**NOX1 expression regulates pulmonary microvascular endothelial barrier function in response to PLY.** (**A**) Dose-dependent administration of NOX1 adenovirus to HLMVECs results in greater superoxide production (48 h). HLMVECs were transduced with increasing MOIs of an adenovirus encoding NOX1, NOXO1 and NOXA1. *n* = 5. (**B**) HLMVECs were transduced with a control virus (pAD RFP) or pAD NOX1, NOXO1 and NOXA1 at the indicated MOI and transendothelial resistance (TER) was monitored over time on an ECIS array. *n* = 4–6. (**C**) HPAECs were transduced with RFP (3 MOI) or Nox1, NOXO1 and NOXA1 (3 MOI) and 24 h later transendothelial resistance (TER) was monitored in the presence and absence of PLY (30 ng). *n* = 4–6. (**D**) COS-7 cells expressing NOX1, NOXO1 or NOXA1 were treated with increasing concentrations of GKT136901 and superoxide levels were measured by L-012 chemiluminescence. (**E**) HPAECs were pretreated with GKT136901 (3 µM) or the vehicle (DMSO), challenged with PLY (30 ng/mL), and transendothelial resistance was monitored over time via ECIS. *n* = 4. (**F**) HLMVECs were transfected with control or Nox1 siRNA and 24 h later PLY-induced endothelial barrier disruption was assessed. *n* = 4–6. (**G**) HLMVECs were transduced with the control virus (pAD RFP) or pAD NOX4. *n* = 4. Data is expressed as means ± S.E.; * *p* < 0.05 vs. control; # *p* < 0.05 vs. NOX4. (**A**,**D**,**G**) ANOVA, (**B**,**C**,**E**,**F**) Student’s *t*-test.

In previous studies we have shown that the db/db mouse model of obesity results in increased NOX1 expression in endothelial cells [38,39]. To investigate the effects of NOX1 activity and NOX1-derived ROS on endothelial barrier integrity, human lung microvascular endothelial cells (HLMVECs) were transduced with an adenovirus encoding NOX1. To create an active NOX1 enzyme, co-expression of the NOX1 subunits NOXA1 and NOXO1 is also required. Accordingly, references to the transgene expression of NOX1 throughout this study also reflect the co-expression of an equal amount of NOXA1 and NOXO1 unless otherwise specified. Increased expression of NOX1 (and NOXA1 and NOXO1) in HLMVECs resulted in a dose-dependent increase in superoxide production as determined using L-012 chemiluminescence [45] (Figure 3A). In parallel experiments, we assessed the effect on barrier function in human endothelial cells. Human lung microvascular endothelial cells (HLMVECs) were plated on electric cell–substrate impedance sensing (ECIS) arrays, grown to confluency and steady-state resistance. HLMVECs were then exposed to ad NOX1 (at 3 or 10 MOI) or control adenovirus (RFP, 10 MOI). Endothelial barrier integrity was disrupted in a dose-dependent manner as indicated by the decrease in transendothelial resistance (TER) (Figure 3B). To test the effect of increased NOX1 expression on PLY-induced endothelial permeability, human pulmonary artery endothelial cells (HPAECs) were transduced with 3 MOI of either NOX1 or RFP and 24 h later PLY (30 ng/mL) was added to the wells. As shown in Figure 3C, PLY-induced decreases in TER were amplified in the NOX1-treated group vs. the control group. To test whether it was NOX1 activity directly or the NOX1 protein that played a role in this effect, a selective inhibitor of NOX1/NOX4 was given to cells prior to treatment with PLY. We first determined the efficacy of GKT136901 against NOX1-derived superoxide production in a heterogenous expression system in COS7 cells (NOX1, NOXO1 and NOXA1), wherein an approximate IC50 of 3 µM was observed (Figure 3D). Treatment of HPAECs with 3 µM GKT137831 significantly attenuated the decrease in TER following treatment with PLY (Figure 3E). To increase confidence that this effect was a result of NOX1 inhibition rather than off-target effects, HLMVECs were transfected with a non-targeting control or an NOX1-specific siRNA and then later treated with PLY. NOX1 knockdown (KD) showed a significant protective effect against PLY-induced endothelial barrier disruption (Figure 3F). To exclude influence by NOX4, which emits hydrogen peroxide instead of superoxide, we transduced HLMVECs with an NOX4 adenovirus, then measured barrier function followed by treatment with or without PLY. The loss of barrier function in NOX4-expressing HLMVECs in response to PLY was reduced compared to the control (RFP), indicating a major difference from NOX1 (Figure 3G). Additional studies using catalase to scavenge H_2_O_2_ provided further evidence against H_2_O_2_ having a role in the loss of barrier function caused by PLY (Supplemental Figure S2A).

**Figure 4:**
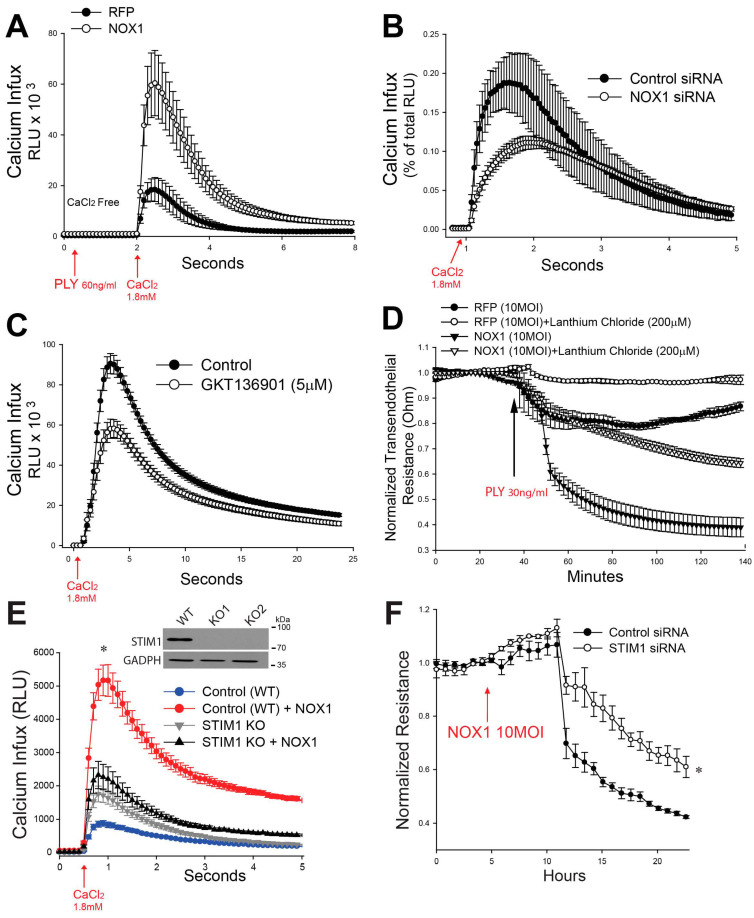
**NOX1 amplifies PLY-induced calcium influx in HLMVECs via activation of STIM1.** (**A**) HLMVECs were transduced with Ad NOX1 vs. a control virus, incubated in Ca^2+^-free media and then stimulated with PLY (60 ng/mL). Calcium influx was monitored following the re-addition of 1.8 mM Ca^2+^. (**B**) Calcium (Ca^2+^) influx was similarly measured in HLMVECs transfected with control or NOX1 siRNA or (**C**) treated with the NOX1 inhibitor (GKT136901). In (**D**), HLMVECs were transduced with Ad RFP or NOX1 in the presence and absence of lanthanum chloride and their barrier function was determined by ECIS. *n* = 4. In (**E**), STIM1 was knocked out in HEK293 cells using CRISPR-Cas9, a WB analysis was carried out, and the cells were stimulated with PLY (60 ng/mL) in Ca^2+^-free media, prior to the addition of Ca^2+^ (1.8 mM). In (**F**), HLMVECs were transfected with control and STIM1 siRNA and then treated with pAD NOX1 and their barrier function was measured over time using ECIS. *n* = 4–6. * *p* < 0.05. (**A**–**C**,**F**) Student’s *t*-test and (**D**,**E**) ANOVA.

We next investigated the mechanisms by which NOX1 regulates intracellular calcium influx. As shown in Figure 4A, increased expression of NOX1 via adenovirus in HLMVECs dramatically increased PLY-stimulated increases in calcium influx. Silencing NOX1 or inhibition with GKT136901 attenuated the ability of PLY to stimulate calcium entry (Figure 4B,C). NOX1-dependent stimulation of calcium influx also contributed to the ability of PLY to stimulate EC barrier dysfunction, as shown by the ability of lanthanum chloride (LaCl3) to block the enhanced decline in barrier function seen in NOX1-transduced HLMVECs (Figure 4D). To further investigate the relationship between NOX1 and Ca^2+^ influx in PLY-induced endothelial barrier dysfunction, we next investigated STIM1 as a possible signaling mechanism. The rationale being that STIM1 was previously reported to be involved in Ca^2+^ influx in LPS-treated epithelial cells [50,51]. To determine if NOX1 was activating STIM1, a critical intracellular component of store-dependent calcium entry, we generated STIM1 KO HEK293 cells using CRISPR-Cas9 and calcium influx was determined in WT or STIM-1 KO cells with or without NOX1 (plus NOXO1/NOXA1). In WT cells, NOX1 greatly amplified PLY-induced calcium entry and these effects were significantly blunted in cells where STIM1 was absent (Figure 4E). In HLMVECs, STIM1 siRNA significantly attenuated the NOX1-mediated decrease in barrier function (Figure 4F). To further confirm this mechanism and explore a translationally relevant approach, HLMVECs were pretreated with YM-58483 (5 µM), an inhibitor of store-operated calcium entry, and then treated with PLY. Endothelial barrier integrity was measured over time using ECIS. We observed a significant blunting of PLY-induced endothelial hyperpermeability, as indicated by improvements in TER (*p* < 0.05), and a significant decrease in calcium influx (Supplemental Figure S2B,C).

### 3.4. Novel Roles for STIM1 and TRPC1 in Mediating PLY-Induced Calcium Entry in Human Endothelial Cell Monolayers

**Figure 5:**
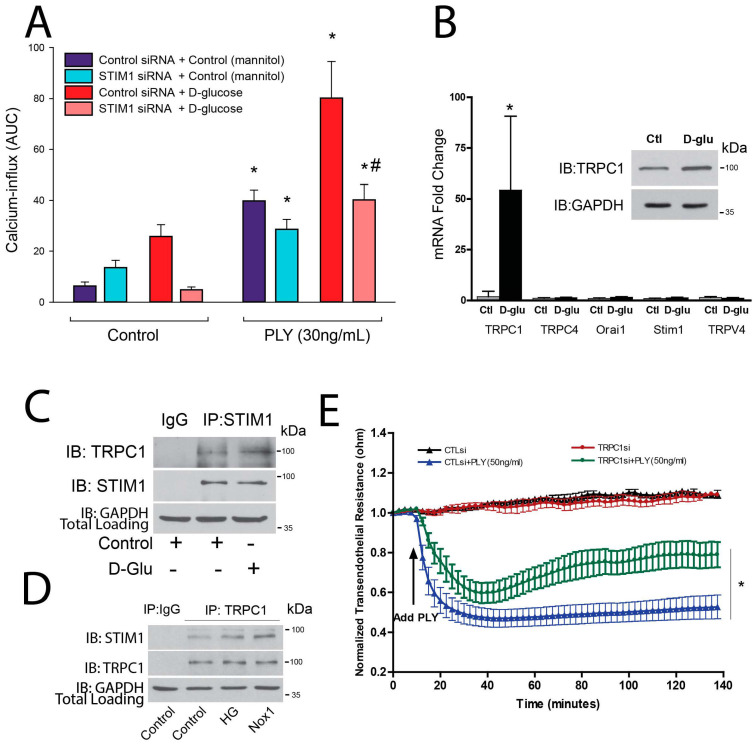
**TRPC1 is the effector of STIM1 signaling in high-glucose conditions.** (**A**) Ca^2+^ influx (pAD AEQ) was measured in HLMVECs 24 h after transfection with control or STIM1 siRNA in response to PLY (30 ng/mL). (**B**) HLMVECs were given control (25 mM mannitol) or high-glucose (25 mM) treatment for 24 h and then cells were collected for RNA and WB analysis. (**Insert**): Western blot of an HLMVEC similarly cultured under control and high-glucose conditions using antibodies to TRPC1 and GAPDH. (**C**) A co-IP experiment was conducted using a control (IGG) or STIM1 antibody for pulldown of HLMVECs given control (25 mM mannitol) or high-glucose (25 mM) treatment for 24 h. (**D**) A co-IP experiment was conducted using a control (IGG) or STIM1 antibody for pulldown of HLMVECs given control (25 mM mannitol) or high-glucose (25 mM) treatment or NOX1 adenovirus for 24 h. (**E**) HLMVECs were transfected with control or TRPC1 siRNA and 24 h later PLY-induced endothelial barrier disruption was assessed. *n* = 4. Data is expressed as means ± S.E.; * *p* < 0.05 vs. control, # indicates *p* < 0.05 vs. PLY and D-Glucose treated control group.; (**A**) ANOVA; (**B**,**E**) Student’s *t*-test.

We next investigated whether targeting STIM1 normalizes PLY-induced calcium influx in hyperglycemic conditions. HLMVECs were treated with PLY, which stimulated calcium influx, and the magnitude of the calcium influx was amplified by preincubation in high-glucose conditions. Selective knockdown of STIM1 in HLMVECs restored glucose-increased calcium influx back to control levels (Figure 5A). To better understand how STIM1 contributes to these changes in calcium, we next investigated the expression of ion channels with known interactions with STIM1 in response to culture in high-glucose conditions. TRPC1, but not Orai1, was found to be elevated at both the transcriptional and protein levels after 24 h in HG conditions (Figure 5B). HG treatment increased the phosphorylation of p38 but did not modify STIM1 protein expression (Supplemental Figure S2D). To investigate whether STIM1 and TRPC1 directly interact in HLMVECs, reciprocal IP experiments were performed (Figure 5C,D), showing that TRPC1 and STIM1 interact directly. Interestingly, we observed that this interaction increases with high-glucose treatment or NOX1 overexpression. To assess the functional role of TRPC1 on endothelial permeability, HLMVECs were transfected with control or siTRPC1 siRNA. Loss of TRPC1 expression in HLMVECs blunted the ability of PLY to disrupt the EC barrier (Figure 5E).

### 3.5. Importance of the Mitochondrial Permeability Transition Pore in PLY-Induced Disruption of the EC Barrier

**Figure 6:**
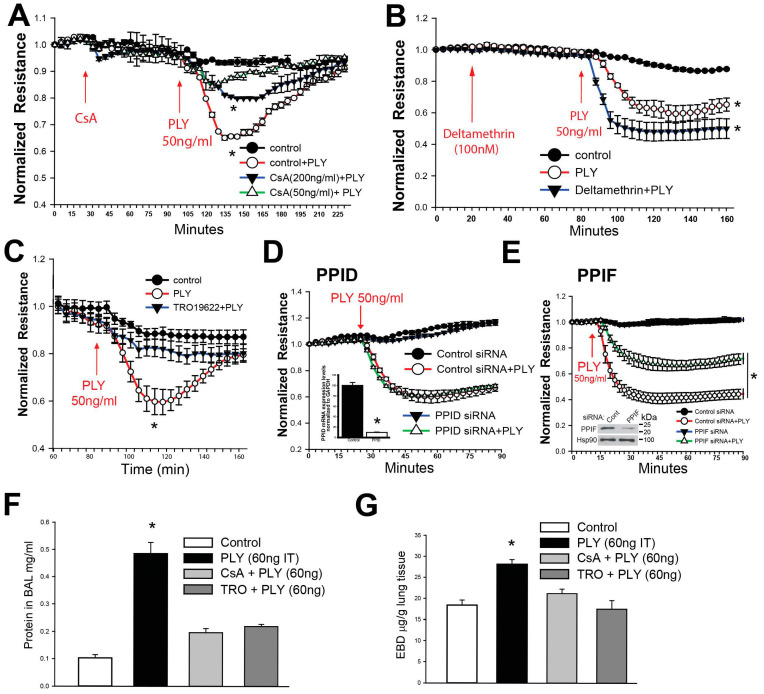
**PLY-induced opening of the mitochondria permeability transition pore (MPTP) leads to a loss of endothelial barrier integrity.** (**A**) HLMVECs were treated with cyclosporine A (CsA, 50–200 ng/mL) followed by PLY (50 ng/mL) and changes in barrier function were determined by ECIS. (**B**) HLMVECs were treated with the calcineurin inhibitor deltamethrin (100 nM), followed by PLY (50 ng/mL). In (**C**), HLMVECs were pretreated with the MPTP inhibitor TRO19622 (10 µM), followed by PLY (50 ng/mL). In (**D**,**E**), HLMVECs were transfected with siRNA to cyclosporine targets PPID and PPIF and then challenged with PLY (50 ng/mL). (**Insert**) Western blot of an HLMVEC transfected with control or PPIF siRNA using antibodies to PPF and Hsp90 (loading control). In (**F**,**G**), mice were administered CsA (10 µg/kg) and TRO19622 (280 µg/kg) IV 60 min prior to IT PLY (60 ng/mouse). Protein levels in the BAL and EBD (following 30 mg/kg IV) were determined. *n* = 4. Data is expressed as means ± S.E.; * *p* < 0.05 vs. control; ANOVA.

We next investigated the consequences of calcium influx in HLMVECs. The mitochondria are a significant buffer of intracellular calcium and excessive calcium influx can lead to mitochondrial Ca^2+^ overload [52]. One of the consequences of this overload is the opening of the mitochondrial permeability transition pore (mPTP). Previous reports show that when the lung epithelium is exposed to PLY the mPTP is activated [53]. To determine a role for the mPTP in endothelial cells in response to PLY, we first treated cells with cyclosporin A, an inhibitor of mPTP opening. We found that cyclosporin A protected the barrier integrity of HLMVECs following treatment with PLY (Figure 6A). However, cyclosporin A can target both mitochondrial cyclophilins and the phosphatase calcineurin. To rule out the latter, we pretreated cells with deltamethrin, a potent and specific inhibitor of calcineurin [54]. Instead of inhibiting the ability of PLY to induce barrier disruption, we found that it amplified the loss of barrier function (Figure 6B), ruling out calcineurin as the mediator of barrier dysfunction and focusing our study on the mPTP. Therefore, we next used a selective inhibitor of the mPTP (TRO19622) which binds directly to the voltage-dependent anion channel and translocator protein (two components of the mPTP). We found that inhibition of mPTP opening in HLMVECs reduced the ability of PLY to increase endothelial permeability (Figure 5C). We next investigated two members of the Peptidyl-prolyl cis/trans isomerase family that have been associated with mitochondrial-mediated cell death pathways. PPID is not part of the mPTP and its knockdown had no effect on endothelial cell barrier function or TER following PLY induction (Figure 6D). In contrast, PPIF is a major component of the mPTP and its knockdown significantly protected against the loss of endothelial barrier function following PLY treatment (Figure 6E). To test this effect in vivo, mice were treated with mPTP inhibitors (CsA, TRO19622) for 30 min prior to intratracheal injection of PLY. Loss of lung barrier function was determined using levels of protein found in the Bronchoalveolar Lavage (BAL) and Evan’s blue dye extravasation. We found that inhibition of the mPTP with TRO19622 protected against PLY-induced loss of barrier function (Figure 6F,G).

## 4. Discussion

Obesity and diabetes are broadly appreciated to increase susceptibility to many diseases, including cancer and cardiovascular and infectious diseases [55]. More recently, the COVID-19 pandemic revealed the profound impact of obesity on the severity of lung disease and mortality [56]. Obesity is a complex disease and how it impacts lung pathology remains poorly understood. Herein, our goal was to identify novel mechanisms through which obesity and hyperglycemia contribute to the loss of pulmonary barrier function. We found that in a mouse model of obesity and T2D, the ability of PLY to induce lung barrier dysfunction was increased and that HG treatment amplified PLY-induced barrier disruption and calcium entry in an NOX1-dependent manner. NOX1-derived ROS promoted the loss of endothelial cell barrier function and increased the barrier-destructive effects of PLY via enhanced calcium entry. We identified an important functional role for STIM1 and its interaction with TRPC1 in mediating enhanced calcium entry and also found that elevated intracellular calcium is associated with opening of the mitochondrial permeability transition pore.

NOX1 is an enzyme linked to a number of disease pathologies including cancer [57], inflammatory bowel disease [58] and cardiovascular disease [29,59]. Our research group has previously identified that NOX1 is upregulated in endothelial cells in the db/db mouse model of obesity/T2D and that NOX1 contributes to endothelial cell dysfunction [38,39,60]. However, the role of NOX1 in regulating endothelial barrier function during pneumonia remains poorly understood. Important roles for NOX1 in regulating barrier function have been shown in the blood–brain barrier [61,62,63] and the intestinal epithelium [64] but relatively little is known concerning the lung [65]. Although we recently reported on an important role for NOX2 in pneumococcal pneumonia-associated lung injury [48], no study to date has looked at the connection between pneumococcal PLY and NOX1 in the lung endothelium in the context of obesity or elevated glucose. Given the ability of NOX1 inhibitors to attenuate PLY-induced lung barrier disruption, it is likely that the NOX1-derived ROS and not the NOX1 protein per se are causative. Thus, other sources of ROS in the endothelium, beyond that produced by NOX1, are likely to function similarly. To this end, we investigated whether NOX4, which functions differently than NOX1 by emitting hydrogen peroxide (H_2_O_2_) instead of superoxide (O_2_^−^), can drive the loss of endothelial barrier function. We found that NOX4 may instead have a barrier-protective function, which suggests that O_2_^−^, and not H_2_O_2_, is promoting the loss of barrier function. Furthermore, catalase, an antioxidant that scavenges H_2_O_2_, but not O_2_^−^, did not affect the ability of NOX1 to drive barrier disruption, providing further evidence that O_2_^−^, but not H_2_O_2_, is barrier-disruptive. Our prior studies showed an important role for NADPH oxidase 2 (NOX2) in lung capillary endothelial cell barrier dysfunction in PLY-treated mice [48], but it is not yet known whether NOX2 has an important role under hyperglycemic conditions. We have shown that obesity/T2D increases NOX1, along with NOXO1 and NOXA1, but not NOX2 [60]. Mitochondria-derived ROS are known to be elevated by HG conditions and increased in obesity [66], but whether they have a role in PLY-induced barrier disruption has not been described. In neurons, PLY has been shown to act similarly, causing rapid increases in reactive oxygen species and calcium followed by mitochondrial damage [67]. Our study advances a pathway through which HG-induced ROS facilitate calcium entry and the opening of the mPTP.

Calcium influx is a major mechanism through which PLY disrupts function in epithelial cells [68,69], platelets [70], and endothelial cells [8]. Our studies in endothelial cells using lanthanum chloride show that blockade of calcium entry through calcium channels protected the endothelial barrier, suggesting that calcium entry through the large plasma membrane pores formed by PLY does not drive bulk calcium entry, but instead stimulates calcium entry via other mechanisms, including store-operated calcium channels. Given the unimpeded flux of ions through PLY membrane pores, this suggests that the number, and perhaps size, of membrane pores assembled by PLY is insufficient to drive sustained calcium entry and/or that the pores are quickly repaired or removed. It is important to also emphasize that the amount of PLY can have profound impacts; wherein lower doses increase calcium levels and intracellular signaling and high doses can cause cell lysis and death. Our results should therefore be interpreted with the perspective that the involvement of the identified pathways will be of greater consequence at lower concentrations of PLY and of diminishing importance with higher doses. There have been some reports of roles for the STIM1 pathway in *Streptococcus pyogenes* toxin-induced injury [69] and ventilator-induced endothelial injury [71], but our study presents the first evidence for a contributing role of STIM1 in endothelial barrier disruption in response to PLY and under conditions of elevated superoxide and hyperglycemia. We found that high-glucose conditions increased the expression levels of TRPC1 in human lung endothelial cells. It has previously been reported that high-glucose conditions can increase TRPC1 in bovine aortic endothelial cells and enhance calcium entry [72], but the effect of high-glucose conditions on TRPC1 expression is not universal and is cell-dependent [73]. We found that in human lung endothelial cells, STIM1 interacts with TRPC1 and this interaction is increased by high-glucose conditions and elevated ROS. This ability of STIM1 to bind TRPC1 [74] to regulate calcium entry has been shown by several groups, but there is little information on the impact of high-glucose conditions on this interaction. It is possible that simply the increased expression of TRPC1 in HG conditions is responsible for the increased binding, or that ROS-driven post-translational modifications or changes in subcellular location are important. A limitation of our study is that we did not investigate the specific mechanisms downstream of ROS that may lead to this interaction and the heightened calcium influx. Direct oxidation of channels by ROS has been reported for inositol 1,4,5-trisphosphate receptors, ryanodine receptors, and the sarco/endoplasmic reticulum Ca^2+^ ATPase pump [75]. This increases the possibility that TRPC1 or STIM1 may be directly oxidized by ROS but we did not investigate this further. The identification of the specific residues oxidized by ROS to promote increased STIM1-TRPC1 activity is of interest but beyond the scope of this research.

Opening of the mPTP decreases mitochondrial function and if sustained can result in cell death [76]. Opening of the mPTP may account for some of the cell death that occurs in response to higher concentrations of PLY and, as we have shown, inhibition of the mPTP can preserve endothelial barrier function in vitro and in vivo. High-glucose conditions can promote opening of the mPTP through multiple pathways including ROS, elevated calcium, and hyperpolarization. A limitation of our study is that while PLY can open the mPTP, the respective contributions of NOX1-derived ROS and STIM1/TRPC1 to this remain undefined and whether these effects are amplified in HG conditions remains to be determined.

Our findings may be relevant to the altered actions of other bacterial agents under hyperglycemic conditions, including the pore-forming toxins streptolysin and listeriolysin-O (LLO) and the Gram-negative endotoxin LPS, all of which show similar increases in Ca^2+^ signaling [51,77,78]. These similar mechanisms indicate that other bacterial pathogens may rely on ROS generation in target cells to decrease endothelial barrier integrity. STIM1 in particular has been reported to be involved in LPS-mediated endothelial injury, but whether ROS drive changes in LPS-mediated Ca^2+^ influx has not been investigated [79]. Elevated ROS are also observed in non-bacterial diseases such as COVID-19 and influenza, which also are accompanied by increases in lung endothelial barrier permeability, but whether this occurs through STIM1-ROS-mPTP remains open for investigation [75,80]. A limitation of our studies is that we focused on the mechanisms downstream of the toxin from Streptococcus pneumoniae. High-glucose conditions may also impact the levels of bacteria and thus the amount of toxin released.

## 5. Conclusions

In summary, the data herein advances the importance of the interactions between obesity, high glucose levels, elevated ROS, Ca^2+^ signaling, and mitochondrial function, providing a novel appreciation of the mechanisms leading to increased risk in patients with T2D. Our findings present new opportunities to therapeutically target novel disease-causing pathways such as NOX1, STIM1 and mPTP to mitigate the cellular dysfunction and death that contributes to the loss of endothelial barrier integrity in the setting of pneumonia and potentially to other infectious agents that cause acute lung injury.

## Data Availability

The original contributions (Western blots) presented in this study are included in the Supplementary Material. There are no informatics or omics data sets. Further inquiries can be directed to the corresponding authors.

## Supplementary Materials

The following supporting information can be downloaded at https://www.mdpi.com/article/10.3390/antiox15030275/s1, Figure S1. WB of protein expression. (A). Transduction of HLMVECs with Nox1 adenovirus (MOI indicated) results in increased NOX1 expression. (B). HLMVECs were transfected with control or Nox1 siRNA and 72 h later protein expression was examined via WB. (C). HLMVECs were transfected with control or STIM siRNA and 24 h later NOX1 adenovirus-induced protein expression was assessed via WB. Figure S2. (A). HLMVECs were treated with D-glucose (25 mM) or an osmotic control (ctl, mannitol 25 mM) for the indicated times and Western blots were performed using antibodies to phosphorylated PO4-38, STIM1, and GADPH. (B). HLMVECs were pretreated with the CRAC inhibitor YM-58483 and then stimulated with PLY (30 ng/mL) and their barrier function was determined by ECIS. In (C), calcium influx was measured in PLY-stimulated HLMVECs in +/− YM-58483. Cells were incubated in calcium-free media and stimulated with PLY (60 ng/mL). Calcium influx was monitored following the re-addition of 1.8 mM calcium. *n* = 4–5. * *p* < 0.05. (D). TER recorded over time in HLMVECs transduced with control or Nox1 adenovirus (10MOI) along with NOXO1 and NOXA1 in the presence of a vehicle or catalase (100 U/mL). *n* = 4–6, * *p* < 0.05. Student’s *t*-test.

## Abbreviations

The following abbreviations are used in this manuscript:

EC	Endothelial Cell
ROS	Reactive Oxygen Species
PLY	Pneumolysin
mPTP	Mitochondrial Permeability Transition Pore
HG	High Glucose
